# Supplementary material for: Perceptual decisions and oculomotor responses rely on temporally distinct streams of evidence
Source: Commun Biol. 2022 Mar 1;5:189. doi: 10.1038/s42003-022-03141-1 (PMC8888581; doi:10.1038/s42003-022-03141-1)
Supplement: Supplementary file 2 — Supplementary Material [file 42003_2022_3141_MOESM2_ESM.pdf]

Tables and Figures Supplementary to:

Perceptual decisions and oculomotor responses rely on temporally distinct streams of evidence

Matteo Lisi[123*]

Michael J. Morgan[1]

Joshua A. Solomon[1*]

[1] Centre for Applied Vision Research, City, University of London, UK

[2] Department of Psychology, University of Essex, Colchester, UK

[3] Department of Psychology, Royal Holloway, University of London, Egham, UK

[*] Corresponding authors: matteo.lisi@rhul.ac.uk, j.a.solomon@city.ac.uk

Table S1. **Summary performance statistics for individual observers (Experiment 1).** The first column indicates the mean distance between the centers of the two generative distributions of target positions averaged across all sessions. Mean response times (third column) are reported with standard deviations within parentheses. The last column indicates how many trials were included in the analysis after the exclusion criteria reported in the main text.

	**Mean distance difference**	**Frequency correct**	**Response time**	**N. trials included in analysis**
sj101	1.25 deg	0.74	605 (172) ms	974
sj102	1.26 deg	0.74	631 (159) ms	1604
sj103	1.09 deg	0.74	610 (149) ms	3135
sj104	0.92 deg	0.81	723 (162) ms	860

Table S2. **Summary performance statistics for individual observers (Experiment 2).**

	Mean luminance difference	Frequency correct	Response time	N. trials included in analysis
sj201	2.73 cd/m^2	0.82	634 (156) ms	477
sj202	2.83 cd/m^2	0.77	727 (124) ms	491
sj203	1.99 cd/m^2	0.80	739 (125) ms	937
sj204	1.63 cd/m^2	0.71	685 (117) ms	788
sj205	1.52 cd/m^2	0.75	720 (129) ms	484

Table S3. **Summary performance statistics for individual observers (Experiment 3).**

	Mean distance difference	Frequency correct	Response time	N. trials included in analysis
sj301	0.88 deg	0.76	644 (144) ms	907
sj302	0.82 deg	0.72	544 (137) ms	1041
sj303	0.91 deg	0.74	675 (155) ms	658
sj304	0.99 deg	0.71	446 (116) ms	977

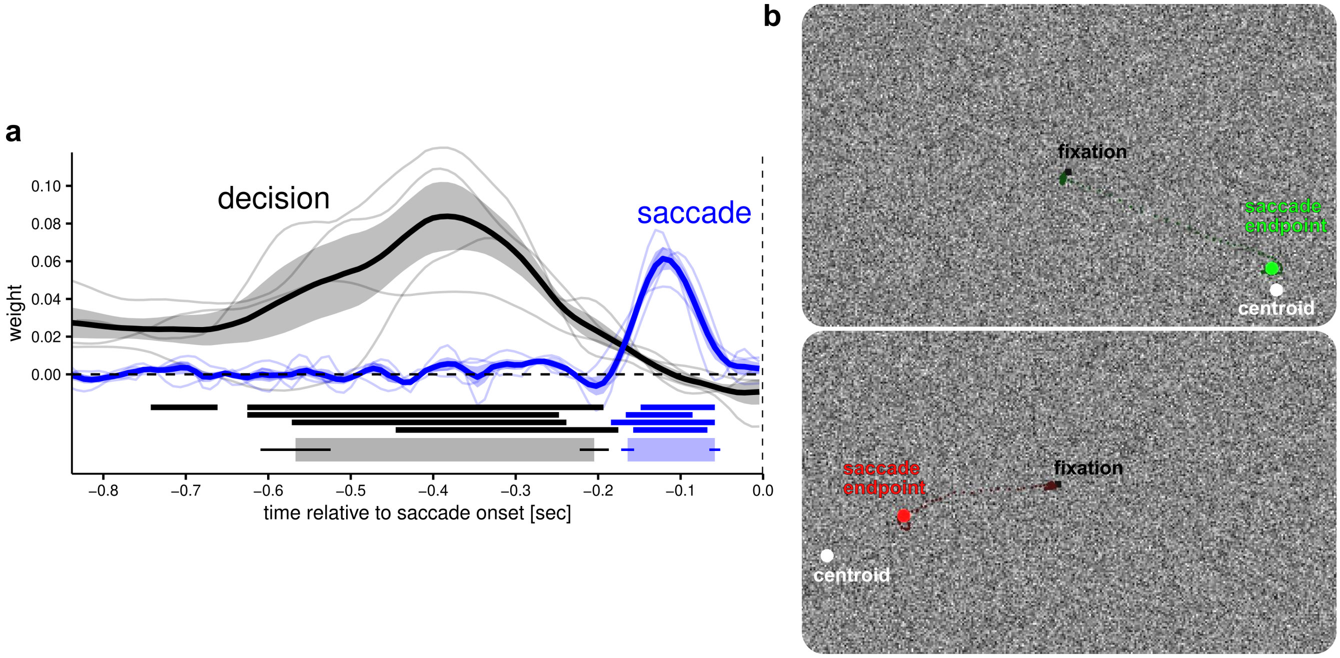

Figure S1. **Experiment 3.** Estimated weighting functions (panel **a**), same conventions as Figure 1 (main text). Panel **b** represents two examples of the feedback. Both the top and the bottom sub-panels represent trials in which the observers made the correct perceptual decision, as indicated by the fact that saccadic endpoints (large green and red dots) are on the same side of fixation as the revealed centroids (white dots). However, on the top sub-panel, the saccade endpoint is colored in green as it was less than 1.25 deg away from the centroid, whereas on the lower sub-panel it is colored in red to indicate the subject that their saccade should have been more accurate. On both sub-panels the smaller, darker green and red dots show all gaze samples from target onset to saccade completion. Text labels are shown for illustration purposes but were not shown during the experiment.

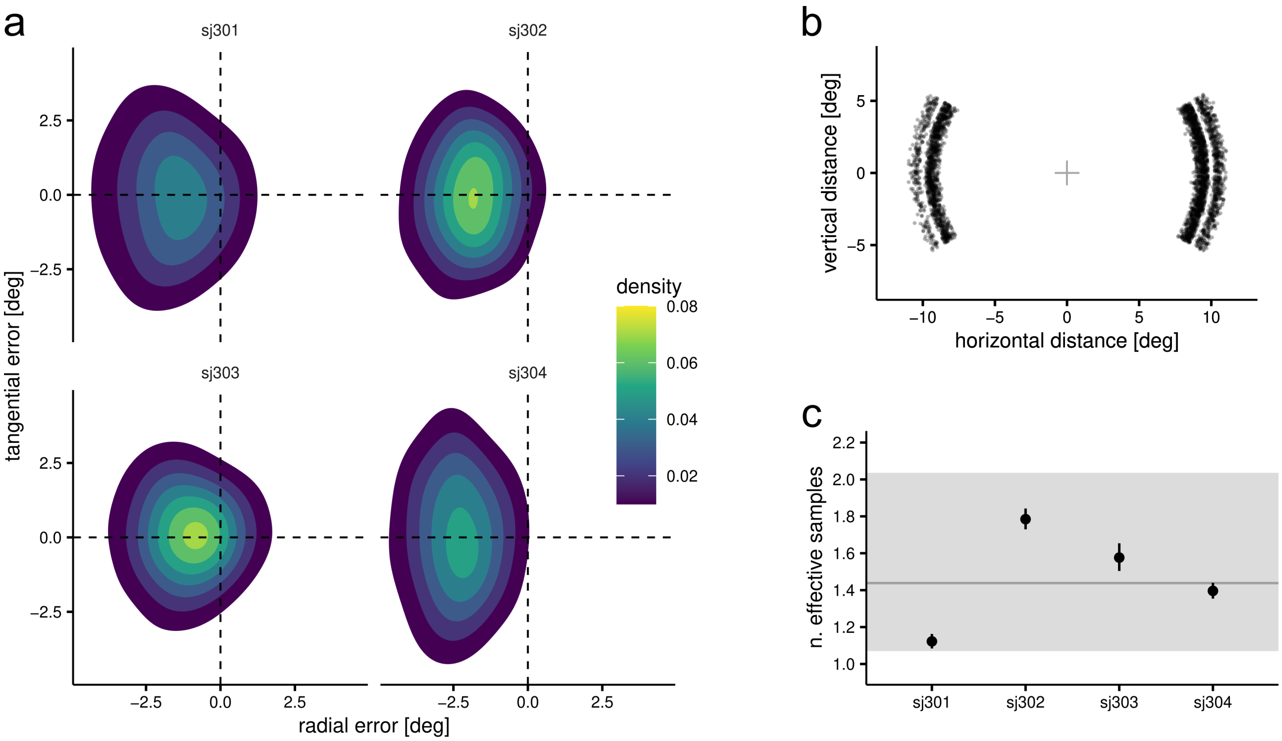

Figure S2. Saccadic precision in Experiment 3. Each facet of panel **a** shows the distribution of saccadic errors relative to the centroid of the generative distribution. These centroids were sampled from iso-eccentric semicircles, whose eccentricities (panel **b**) were determined by the staircase procedure. We attempted to estimate the effective number of blob samples averaged by the observers to localize the centroid based on data from Experiment 1 of Lisi, et al. (2019), which used the same visual stimulus (a Gaussian blob with same space constant, peak luminance, eccentricity, and noise background), but as single, static, target for the observers' saccades. If we assume that the dominant noise in both experiments independently affected the location of each blob, then the effective sample size in the current Experiment 3 would be

$$N = \frac{\sigma^2_{\text{single}} + (1.5\ \text{deg})^2}{\sigma^2}$$

where σ^2_{single} represents the variance of saccadic errors for the single targets (as measured in Lisi, et al., 2019), 1.5 deg is the standard deviation of blob positions in our current experiment, and σ^2 is the saccadic variance measured in the current Experiment 3. One complication concerns the dimensionality of saccadic variance. Whereas centroid eccentricity was fairly stable in the current Experiment 3, the target's direction with respect to fixation varied along two semi-circles (as shown in panel **b**). In Lisi, et al. (2019), the situation was reversed: the target always appeared on the horizontal meridian, but its eccentricity varied (between 8 and 10 deg). Thus, to fairly compare performances across these two studies, we used averages of saccadic variance in the radial and tangential dimensions for both σ^2_{single} and σ^2. Panel **c** shows the estimated sample size *N* for each observer (black dots; error bars are standard errors), as well as the average across observers (grey horizontal line; the error band represents 95% confidence interval). These estimated sample sizes agree nicely with the 106-ms duration of saccadic integration windows in Experiment 3, which corresponds to approximately 1.6 blobs. Note, however, these estimates of effective sample size should be understood as a lower bound. If we were to relax our

assumption regarding the dominance of early noise, and acknowledge the non-negligible effects of late noise (including motor noise) and calculation inefficiencies, then the true sample size used by participants would be larger than these estimates.
