# Supplementary material for: Perceptual decisions and oculomotor responses rely on temporally distinct streams of evidence
Source: Commun Biol. 2022 Mar 1;5:189. doi: 10.1038/s42003-022-03141-1 (PMC8888581; doi:10.1038/s42003-022-03141-1)
Supplement: Supplementary file 3 — Description of Additional Supplementary Files [file 42003_2022_3141_MOESM3_ESM.pdf]

Description of Additional Supplementary Files

File name: Supplementary Data 1
Description: Estimated weighting functions for Experiment 1 and 2, shown in Fig. 1 (full dataset and analysis code available at https://osf.io/2f7k6/).

File name: Supplementary Data 2
Description: Estimated weighting functions split by latency quartiles, as shown in Fig. 2, and saccadic latency data.

File name: Supplementary Data 3
Description: Data on speed and accuracy, shown in Fig. 3.

File name: Supplementary Data 4
Description: Estimated weighting functions for Experiment 3, shown in Fig. S1.

File name: Supplementary Data 5
Description: Saccadic landings data for Experiment 3, as shown in Fig. S2.
