# Supplementary material for: Perceptual decisions and oculomotor responses rely on temporally distinct streams of evidence
Source: Commun Biol. 2022 Mar 1;5:189. doi: 10.1038/s42003-022-03141-1 (PMC8888581; doi:10.1038/s42003-022-03141-1)
Supplement: Supplementary file 9 — Reporting Summary [file 42003_2022_3141_MOESM9_ESM.pdf]

nature portfolio

Corresponding author(s): Matteo Lisi and Joshua A. Solomon

Last updated by author(s): Jan 8, 2022

Reporting Summary

Nature Portfolio wishes to improve the reproducibility of the work that we publish. This form provides structure for consistency and transparency in reporting. For further information on Nature Portfolio policies, see our Editorial Policies and the Editorial Policy Checklist.

Statistics

For all statistical analyses, confirm that the following items are present in the figure legend, table legend, main text, or Methods section.

n/a	Confirmed	
☐	☒	The exact sample size (*n*) for each experimental group/condition, given as a discrete number and unit of measurement
☐	☒	A statement on whether measurements were taken from distinct samples or whether the same sample was measured repeatedly
☐	☒	The statistical test(s) used AND whether they are one- or two-sided<br>*Only common tests should be described solely by name; describe more complex techniques in the Methods section.*
☐	☒	A description of all covariates tested
☐	☒	A description of any assumptions or corrections, such as tests of normality and adjustment for multiple comparisons
☐	☒	A full description of the statistical parameters including central tendency (e.g. means) or other basic estimates (e.g. regression coefficient) AND variation (e.g. standard deviation) or associated estimates of uncertainty (e.g. confidence intervals)
☐	☒	For null hypothesis testing, the test statistic (e.g. *F*, *t*, *r*) with confidence intervals, effect sizes, degrees of freedom and *P* value noted<br>*Give P values as exact values whenever suitable.*
☐	☒	For Bayesian analysis, information on the choice of priors and Markov chain Monte Carlo settings
☒	☐	For hierarchical and complex designs, identification of the appropriate level for tests and full reporting of outcomes
☐	☒	Estimates of effect sizes (e.g. Cohen's *d*, Pearson's *r*), indicating how they were calculated

Our web collection on statistics for biologists contains articles on many of the points above.

Software and code

Policy information about availability of computer code

Data collection	Data and code supporting this article are available as an Open Science Framework repository (link: https://osf.io/embky/).
Data analysis	Data and code supporting this article are available as an Open Science Framework repository (link: https://osf.io/embky/).

For manuscripts utilizing custom algorithms or software that are central to the research but not yet described in published literature, software must be made available to editors and reviewers. We strongly encourage code deposition in a community repository (e.g. GitHub). See the Nature Portfolio guidelines for submitting code & software for further information.

Data

Policy information about availability of data

All manuscripts must include a data availability statement. This statement should provide the following information, where applicable:

- Accession codes, unique identifiers, or web links for publicly available datasets
- A description of any restrictions on data availability
- For clinical datasets or third party data, please ensure that the statement adheres to our policy

Data and code supporting this article are available as an Open Science Framework repository (link: https://osf.io/embky/).

Field-specific reporting

Please select the one below that is the best fit for your research. If you are not sure, read the appropriate sections before making your selection.

☒ Life sciences ☐ Behavioural & social sciences ☐ Ecological, evolutionary & environmental sciences

For a reference copy of the document with all sections, see nature.com/documents/nr-reporting-summary-flat.pdf

Life sciences study design

All studies must disclose on these points even when the disclosure is negative.

Sample size	All samples are in excess of N = 400. See Table S1.
Data exclusions	As stated in the text, "We excluded trials where the primary saccade had a latency shorter than 100ms (about 0.5% of total trials) and trials where the amplitude of the primary saccade was less than 2 degrees (about 4% of total trials)."
Replication	The statistical significance was tested within each participant at the alpha level of 0.01, thus each participant can be seen as an independent replication.
Randomization	"Samples" here are saccades to one of two targets. Properties of those targets were determined using a random-number generator.
Blinding	Not relevant. Although our methods were sophisticated, our measurements were quite simple. We simply asked participants to look at something and we measured their eye movements. Participants were not divided into different groups.

Reporting for specific materials, systems and methods

We require information from authors about some types of materials, experimental systems and methods used in many studies. Here, indicate whether each material, system or method listed is relevant to your study. If you are not sure if a list item applies to your research, read the appropriate section before selecting a response.

Materials & experimental systems

n/a	Involved in the study	
☒	☐	Antibodies
☒	☐	Eukaryotic cell lines
☒	☐	Palaeontology and archaeology
☒	☐	Animals and other organisms
☐	☒	Human research participants
☒	☐	Clinical data
☒	☐	Dual use research of concern

Methods

n/a	Involved in the study	
☒	☐	ChIP-seq
☒	☐	Flow cytometry
☒	☐	MRI-based neuroimaging

Human research participants

Policy information about studies involving human research participants

Population characteristics	2 authors and 2 naive observers participated in Experiment 1. 6 observers participated in Experiment 2 (2 authors and 4 naive observers). These sample sizes were chosen in accordance with similar psychophysical reverse correlation studies in the literature (see Murray, 2011, for a review). All had normal or corrected-to-normal vision.
Recruitment	We used the Sona recruitment system. This is the Standard Operational Procedure for all experiments run in City's Psychology Department.
Ethics oversight	The protocol of the study received full approval from the Research Ethics Committee of the School of Health Sciences of City University of London.

Note that full information on the approval of the study protocol must also be provided in the manuscript.
